# Influence of water temperature, sedimentation and fecal refrigeration times on the recovery of *Dictyocaulus viviparus* larvae by two diagnostic techniques

**DOI:** 10.1590/S1984-29612026015

**Published:** 2026-05-25

**Authors:** Bruno de Oliveira Telles Ferreira, Lais Sperandio Cassani, Ygor Henrique da Silva, Barbara Rauta de Avelar, Manoella Dantas Medeiros, Alyne Mendonça Lima, Laritssa Andrade Pinheiro Magalhães, Thaís Ribeiro Correia

**Affiliations:** 1 Universidade Federal Rural do Rio de Janeiro - UFRRJ, Instituto de Veterinária - IV, Programa de Residência em Medicina Veterinária (Diagnóstico em Parasitologia Animal), Seropédica, RJ, Brasil; 2 Universidade Federal Rural do Rio de Janeiro - UFRRJ, Instituto de Veterinária - IV, Programa de Pós-graduação em Ciências Veterinárias, Seropédica, RJ, Brasil; 3 Universidade Federal Rural do Rio de Janeiro - UFRRJ, Instituto de Veterinária - IV, Departamento de Parasitologia Animal - DPA, Seropédica, RJ, Brasil

**Keywords:** Ruminants, diagnosis, coproparasitological examination, dictyocaulosis, Ruminantes, diagnóstico, exame coproparasitológico, dictiocaulose

## Abstract

Bovine dictyocaulosis, caused by *Dictyocaulus viviparus*, is a respiratory infection that leads to economic losses in livestock farming. The objective of this study was to compare two techniques for the diagnosis of *D. viviparus*. Residual fecal samples from routine diagnostics known to be positive were homogenized and subjected to the techniques proposed by modified Rugai, and Ueno techniques. We verified the effects of fecal refrigeration time (+24h and +48h), sedimentation time (2h and 12h), and water temperature (22°C and 46°C) on the recovery and sensitivity of first-stage larva (L1) diagnosis. Fecal refrigeration time (24h vs. 48h) did not significantly affect the number of recovered larvae, demonstrating that samples can be safely stored for up to two days. Room temperature water demonstrated a sensitivity below 75% in the recovery of *D. viviparus* during the first two hours in both techniques, increasing to 100% with 12 hours of sedimentation. However, heating the water to 46ºC proved to be the key factor: larval recovery became equal between the techniques, achieving 100% sensitivity in both after only two hours. We conclude that both techniques have similar sensitivity, making both valid for the recovery and diagnosis of *D. viviparus*.

## Resumo

A dictiocaulose bovina, causada por *Dictyocaulus viviparus*, é uma infecção respiratória que acarreta perdas econômicas na pecuária. O objetivo deste estudo foi comparar duas técnicas para o diagnóstico de *D. viviparus*. Amostras residuais da rotina laboratorial, comprovadamente positivas, foram homogeneizadas e submetidas às técnicas de Rugai modificado e de Ueno. Verificamos os efeitos do tempo de refrigeração fecal (+24h e +48h), tempo de sedimentação (2h e 12h) e temperatura da água (22°C e 46°C) na recuperação e sensibilidade do diagnóstico de larvas de primeiro estágio (L1). O tempo de refrigeração fecal (24h vs. 48h) não afetou significativamente o número de larvas recuperadas, demonstrando que as amostras podem ser armazenadas com segurança por até dois dias. Água em temperatura ambiente demonstrou sensibilidade inferior a 75% na recuperação de *D. viviparus* durante as primeiras duas horas em ambas as técnicas, elevando-se para 100% com 12 horas de sedimentação. Entretanto, o aquecimento da água a 46ºC mostrou-se o fator determinante: a recuperação larval tornou-se equivalente entre as técnicas, atingindo 100% de sensibilidade em ambas após apenas duas horas. Concluímos que ambas as técnicas possuem sensibilidade semelhante, sendo válidas para a recuperação e o diagnóstico de *D. viviparus*.

Bovine dictyocaulosis, caused by the nematode *Dictyocaulus viviparus*, is a severe respiratory infection in cattle. The symptomology varies depending on the animal category affected, characterized by persistent cough, tachypnea, stunted growth, and occasionally sudden death. Responsible for economic losses on dairy and beef cattle, and their occurrence depends on environmental conditions. In Brazil, it’s predominant in the Southern and Southeastern regions, with variable endemicity and prevalence rates, including for the state of Rio de Janeiro. However, there was an increase in cases in recent decades, including outbreaks in adult animals, probably associated with climate change and failures in the activity of anthelmintics based on macrocyclic lactones, benzimidazoles and imidazothiazoles (Campbell et al., 2024, Coelho et al., 2025, Pimentel & Fonseca, 2002; Henker et al., 2017; Macedo et al., 2022). Reinforcing the need for highly sensitive and optimized diagnostic tools to monitor herd health, detect early subclinical infections, and support targeted treatment strategies.

Among the available methods for *in vivo* diagnosis of *D. viviparus* infection, bronchoalveolar lavage, immunodiagnostic techniques, and coproparasitological examinations stand out (Charlier et al., 2016; Danks et al., 2022; Macedo et al., 2022; Sabatini et al., 2023). Coproparasitological examination is the routine, preferred diagnostic method because it is less invasive and more accessible to producers. Among coproparasitological techniques, the Baermann technique and its variations are widely used for larval recovery and morphological identification (Verocai et al., 2020), constituting the principal diagnostic method employed for the detection of first-stage larvae (L1) of *D. viviparus* in feces (Macedo et al., 2022).

Among these variations, the techniques described by Ueno (Ueno & Gonçalves, 1998) and Rugai et al. (1954) are notable, as they establish important adaptations for larval isolation. However, despite various comparisons with the classical Baermann technique, multiple gaps exist. The approaches often require standardizations that prolong sample processing time and rely on utilizing specific equipment that is less accessible for field routines or resources-limited laboratories (Forrester & Lankester, 1997; Verocai et al., 2020).

Although the techniques of Ueno (Ueno & Gonçalves, 1998) and Rugai et al. (1954) optimized the use of material and space (Ueno & Gonçalves, 1998), further comparative validations to reduce processing and diagnostic times are necessary. Accordingly, this study aimed to compare the sensitivity of the two techniques to diagnose *D. viviparus* in bovine fecal samples. Critical variables such as fecal sample refrigeration time, sedimentation time, and water temperature, were examined, aiming for optimization and applicability in different scenarios.

Experimental procedures were performed in the Diagnostic Sector of Animal Parasitology at the Laboratório de Quimioterapia Experimental em Parasitologia Veterinária (LQEPV), in the Departamento de Parasitologia Animal of the Instituto de Veterinária at the Universidade Federal Rural do Rio de Janeiro (UFRRJ).

The study was structured into two sequential stages to evaluate technique performance of the Ueno technique (Ueno & Gonçalves, 1998), which was used as the reference method, and a modified version of the technique described by Rugai et al. (1954).

The experimental design consisted of 12 groups (first stage with eigth groups and second stage four groups) with 36 replicates each, totaling 432 replicates across the entire study. The first stage investigated the effect of fecal sample refrigeration time (24 and 48 hours at 2–8°C) and the larval sedimentation time (2 and 12 hours). The assays were conducted in water at 22 ± 1°C, resulting in eight experimental groups. The second stage aimed to evaluate the impact of water temperature (22 ± 1°C vs. 46 ± 1°C) on larval recovery. For this analysis, the sample refrigeration time was standardized to 24 hours and the sedimentation time was set at 2 hours. The 2 hour period was selected because it was the maximum duration during which the heated water remained above ambient temperature before reaching thermal equilibrium with the laboratory environment. This stage comprised four experimental groups.

The experimental groups were designated by an alphanumeric code representing the technique (U for Ueno, R for modified Rugai), followed by refrigeration time (24 or 48 hours) and sedimentation time (02 or 12 hours), e.g., U2412 or R4802. For the second phase, the code represents the technique and water temperature (e.g., U22 or R46).

The fecal samples used for the study were selected from the routine laboratory of the diagnostic sector, received in an isothermal box at 8°C and been processed no more than 2 hours after receipt for the Ueno & Gonçalves (1998) technique and testing positive with at least 10 larvae after 12 hours of sedimentation. For each phase, approximately 500 grams of feces from five animals were selected according to the criteria described above, and nine replicates were performed from each group until a total of 36 replicates were completed. Thus, feces collected directly from the rectal ampulla of 40 crossbred Holstein calves approximately 15 months old were used.

**Figure 1:**
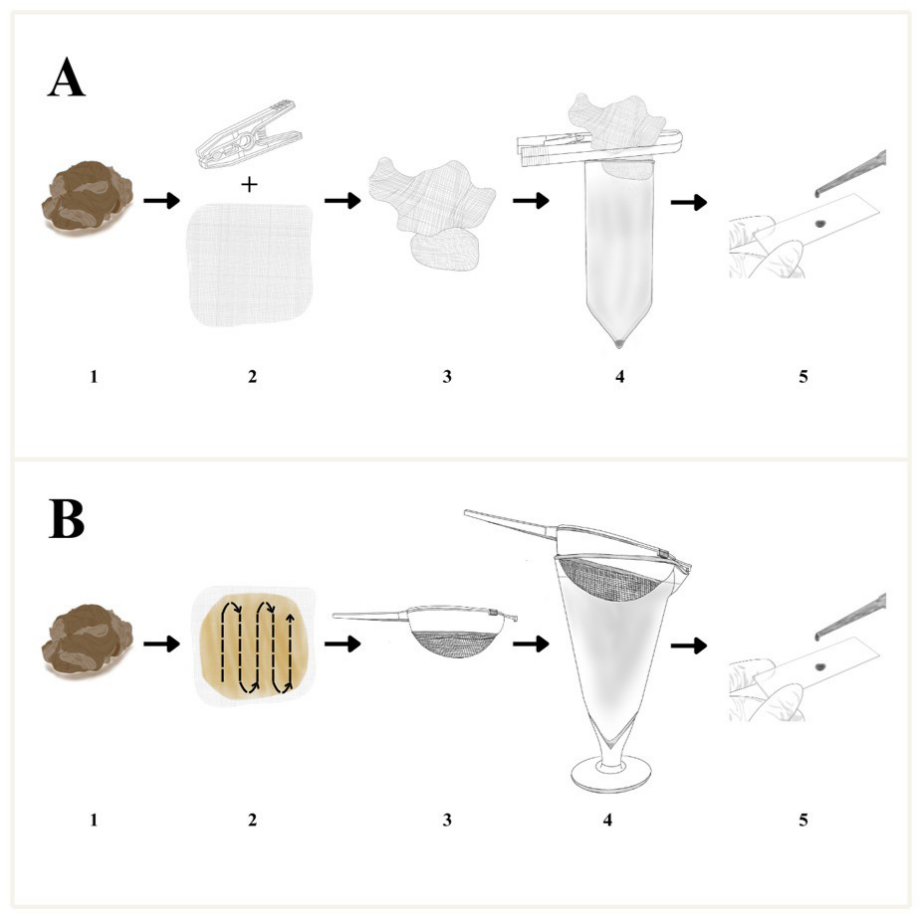
Schematic flowcharts of the diagnostic techniques methodologies. (A) Ueno technique: 2 g of feces (1) are placed in a gauze compress (2) to form a pouch (3), which is submerged in a conical tube containing water (4). After the sedimentation time, 1 mL of the sediment is collected for microscopic analysis (5). (B) Modified Rugai et al. technique: 10 g of feces (1) are spread over gauze (2) supported by a sieve (3), which is placed over a sedimentation flask containing water (4). After the sedimentation time, 1 mL of the sediment is collected for microscopic analysis (5).

Regarding the coproparasitological techniques used, the technique of Ueno & Gonçalves (1998) was performed by weighing 2 g of feces onto a hydrophilic gauze compress (7.5 cm x 7.5 cm, 9 threads/cm^2^), which, after being folded to form a pouch, was submerged in a conical glass tube with filtered water for sedimentation (Figure 1A). While the modified technique of Rugai et al. (1954) involved distributing 10 g uniformly over the surface of a gauze compress with the same specifications (7.5 cm x 7.5 cm, 9 threads/cm^2^), this gauze was positioned on a 100 mesh stainless steel sieve (149 µm opening), and this assembly was placed on a 300 mL glass beaker filled with filtered water until the gauze was submerged for sedimentation (Figure 1B).

At the end of the sedimentation period for each technique, the apparatus containing the fecal sample (gauze bag or sieve) was carefully removed, and a 1 mL aliquot of the sediment was aspirated directly from the bottom of the container using a precision pipette. This was because live, active first-instar larvae of *D. viviparus* concentrate at the bottom due to gravity and positive thermotropism (Ueno & Gonçalves, 1998).

This 1 mL aliquot was transferred to a microscope slide and examined under light microscopy at 40x and 100x magnifications. The larval density, expressed in larvae per gram of feces (LPG), was calculated by directly dividing the total number of larvae counted in this 1 mL of sediment by the initial fecal mass of the aliquot (2 g or 10 g), assuming complete larval sedimentation.

A total of thirty-six repetitions per group were used to detect significant differences between the techniques. All statistical inferences were performed using R software (version 4.4.1), adopting a significance level of α = 0.05. Initially, histograms were generated to evaluate the distribution and normality for each group. The LPG data showed neither normal distribution nor homogeneity of variance. Therefore, the non-parametric Kruskal–Wallis test was used to compare the medians between groups. *Post hoc* analyses were conducted using Dunn’s test with *p*-value correction using the Bonferroni method. In the qualitative data, the diagnostic sensitivity of each group was calculated as the proportion of positive replicates. Differences in the frequencies of positive and negative results between groups were evaluated using Pearson’s Chi-square test (*χ*^2^). The expected frequencies for each cell in the contingency table were calculated by multiplying the respective row total by the column total and dividing by the total of observations. This was followed by the analysis of adjusted standardized residuals to identify the categories that contributed most to any observed statistical significance.

LPG analysis demonstrated that sedimentation time had the greatest impact on larval recovery rate. The Kruskal–Wallis test revealed statistically significant differences between the groups (*p* < 0.0000000209). Dunn’s *post hoc* test indicated that the groups subjected to 2 hours of sedimentation (U2402, R2402, U4802, R4802) did not differ statistically from each other, but presented significantly lower larval recovery compared to the 12-hour groups (Table 1).

Although the U2412 group presented the highest absolute mean larval recovery (20.08 ± 20.45 LPG), statistical analysis revealed no significant difference among the groups subjected to 12 hours of sedimentation (U2412, R2412, U4812, and R4812) (Table 1). Therefore, both techniques combined with a 12 hour sedimentation period yielded statistically equivalent larval recoveries, regardless of whether the samples were refrigerated for 24 or 48 hours.

The qualitative sensitivity analysis corroborated the importance of sedimentation time (Table 1). The Chi-square test indicated a significant association between the method and frequency of positive/negative results (*p* <0.000000002066). Analysis of the adjusted standardized residuals revealed that all groups with 12 hours of sedimentation (U2412, R2412, U4812, R4812) did not differ statistically from each other, reaching 100% or close to sensitivity (97.22% for U4812). In contrast, the groups with two hours of sedimentation showed a significantly higher frequency of negative results than expected, resulting in notably lower sensitivities, ranging from 61.11% (U4802) to 75% (R2402).

**Table 1:** Influence of fecal refrigeration and sedimentation times on the recovery (LPG), expected/observed positive frequencies, and diagnostic sensitivity for *D. viviparus*.

**Sample refrigeration time**	**Technique**	**Sedimentation time**	**Number of larvae identified per gram of feces**			
			**Mean ± SD**	**Median (Min - Max)**	**Positives Obs. (Exp.)** *	**Sensitivity (%)**
**24 hours**	Ueno	2 hours	0.86 ± 1.03^a^	0.5 (0.0 - 4.5)	24 (30.1)	66.67
		12 hours	20.08 ± 20.45^bc^	14.75 (0.5 - 86.5)	36 (30.1)	100.00
	Modified Rugai et al.	2 hours	0.29 ± 0.42^a^	0.2 (0.0 - 2.3)	27 (30.1)	75.00
		12 hours	9.17 ± 5.84^b^	7.9 (0.3 - 27.3)	36 (30.1)	100.00
**48 hours**	Ueno	2 hours	0.71 ± 0.77^a^	0.5 (0.0 - 2.5)	22 (30.1)	61.11
		12 hours	14.46 ± 11.29^c^	11.0 (0.0 - 62.0)	35 (30.1)	97.22
	Modified Rugai et al.	2 hours	0.62 ± 0.74^a^	0.45 (0.0 - 3.2)	25 (30.1)	69.44
		12 hours	6.59 ± 4.99^bc^	5.65 (0.1 - 25.4)	36 (30.1)	100.00

* Exp.: Expected frequencies calculated for the Chi-square test.

SD: Standard Deviation. Different superscript letters in the "Mean ± SD" column indicate statistically distinct groups according to the Kruskal-Wallis test followed by adjusted Dunn’s post hoc test (p < 0.05).

Increasing the water temperature to 46 ± 1°C demonstrated a marked and positive effect on larval recovery when the sedimentation time was fixed at 2 hours (Table 2). Both techniques, Ueno (U46) and modified Rugai et al. (1954) (R46), showed a mean LPG recovery significantly higher (3.01 ± 1.96 LPG and 4.74 ± 2.52 LPG, respectively) compared to their counterparts using water at 22 ± 1°C, U22 (0.71 ± 0.97 LPG) and R22 (0.87 ± 1.22 LPG) (*p* < 0.00000000000000022).

Notably, no statistical difference in larval recovery was observed between the U46 and R46 techniques (Table 2), indicating that, under heating, the techniques have equivalent quantitative performance.

**Table 2:** Influence of water temperature on the recovery (LPG), expected/observed positive frequencies, and diagnostic sensitivity for *D. viviparus* after 2 hours of sedimentation.

**Sample refrigeration time**	**Technique**	**Temperature (°C)**	**Number of larvae identified per gram of feces**			
			**Mean ± SD**	Median (Min - Max)	Positives Obs. (Exp.)*	Sensitivity (%)
**24 hours**	Ueno	22 ± 1	0.71 ± 0.97^a^	0.5 (0.0 - 5.5)	26 (32.2)	72.22
		46 ± 1	3.01 ± 1.96^b^	3.0 (0.5 - 11.0)	36 (32.2)	100.00
	Modified Rugai et al.	22 ± 1	0.87 ± 1.22^a^	0.8 (0.0 - 7.2)	31 (32.2)	86.11
		46 ± 1	4.74 ± 2.52^b^	4.3 (0.4 - 15.4)	36 (32.2)	100.00

* Exp.: Expected frequencies calculated for the Chi-square test.

SD: Standard Deviation. Different superscript letters in the "Mean ± SD" column indicate statistically distinct groups according to the Kruskal-Wallis test followed by adjusted Dunn’s post hoc test (p < 0.05).

In terms of sensitivity, increasing the water temperature to 46 ± 1°C improved the performance of both techniques to 100% (Table 2). The Chi-square test was significant (*p* < 0.0001359) and the analysis of residuals confirmed that both groups (U46 and R46) treated with heated water had a significantly higher frequency of positive results than expected. In contrast, the U22 technique showed a tendency towards negative results, with a sensitivity of only 72.22%.

As secondary findings, unembryonated eggs of the order Strongylida were frequently observed during microscopic analysis. They were detected in approximately 100% of the samples, with specific exceptions of negative samples in groups U4802 (eight) and R2402 (six) and one negative sample in each of the groups U2402, U22, U46, and R4812. First-stage larvae (L1) of Strongylida were not detected during the study. Other important findings, albeit at a lower frequency, included eggs of *Moniezia* spp. and *Trichuris* spp. and cysts of *Buxtonella* spp.

Studies comparing coproparasitological diagnostic techniques have demonstrated possible differences in the sensitivity and/or specificity of certain techniques for specific parasite groups, allowing for a better understanding and generating more reliable results (Verocai et al., 2020). Although the Ueno (1968) and Rugai et al. (1954) techniques have already been validated against the Baermann technique, some gaps remain, as these techniques are often tested and used to evaluate the larval stages of other nematodes.

The use of stored feces is contraindicated by some authors, as storing feces for long periods can lead to the hatching of gastrointestinal nematode eggs (e.g., eggs of the order Strongylida) or the mortality of *D. viviparus* larvae, leading to false-positive or false-negative diagnoses, respectively (Ueno & Gonçalves, 1998; Rode & Jørgensen, 1989; Zajac et al., 2021; Sabatini et al., 2023). However, in the first phase of this study, when we compared the sample refrigeration and sedimentation times, our statistical analysis demonstrated that the refrigeration time (24h vs. 48h) did not significantly affect the number of recovered *D. viviparus* larvae (LPG) nor the diagnostic sensitivity. Thus, as demonstrated by the results in Table 1, extending the refrigeration up to 48 hours does not compromise the diagnosis of *D. viviparus*. Although traditionally, for the diagnosis of *D. viviparus*, the shorter the storage time of feces, the greater the recovery of larvae. These results highlight a highly relevant practical application: in special situations where sample processing cannot be performed immediately, such as properties far from the diagnostic laboratory or a high number of samples to be processed, stored for up to 48 hours at 2–8°C does not generate false positive results. Furthermore, in animals with a low parasite load, extending the sedimentation time to 12 hours reduces the probability of false negatives.

A strong time dependent effect on LPG count and technique sensitivity was verified, demonstrating that the length of time (12 hours) corresponds with the higher number of recovered larvae and sample sensitivity (≥ 97.22%). Previous studies on lungworm diagnosis using the Baermann technique and its variations showed sedimentation times ranging from 8-24 hours (Zajac et al., 2021). Although the sensitivity for *D. viviparus* ranges from 7.4% to 100% depending on the infection period, climatic conditions, and host acquired immunity (Rugai et al., 1954; Lurier et al., 2018), the techniques in this study proved effective for these helminths detection. However, we observed that increasing the sedimentation time also increased the variation in the number of larvae recovered in both techniques. The variation in the LPG value in this case may be related to biases such as sample homogenization, the contact area of ​​the sample with the water, and the retention of L1 in the mesh of the sieves used for the modified technique of Rugai et al. (1954) when using water at 22° ± 1°C.

Water temperature was associated with the number of L1 diagnosed and, consequently, short-term sample diagnostic sensitivity (Table 2). Ueno & Gonçalves (1998), while using the Baermann technique, heated the water to 40°C–50°C and shortened the reading period of *D. viviparus* diagnosis to 1–2 hours. Hernández-Chavarría & Avendaño (2001) used water heated to 37°C in their Baermann technique modification to diagnose *Strongyloides stercoralis*, reducing the sedimentation time to 2 hours while maintaining sensitivity. The temperature increase likely stimulated increased motility in the fecal medium, allowing L1 migration and subsequent sedimentation (Rode & Jorgensen, 1989).

Reported fecal volume varies widely across the literature, ranging from 2–50 g (Lurier et al., 2018; May et al., 2018; Ueno & Gonçalves, 1998). Despite the differences in fecal volume used in each technique (2 or 10g), both the Ueno (Ueno & Gonçalves, 1998) and modified Rugai et al. (1954) techniques allowed us to reach a diagnosis with similar sensitivities. Eysker (1997) explained this by stating that technique sensitivity is high only for primary infections; in older animals, sensitivity is reduced because the large volume of feces causes larval dilution in this medium, although *D*. *viviparus* infections occur mainly in animals up to one year old, and older animals are more resistant to infection, severe cases in adult cattle have increased in the last decade (Henker et al., 2017; Ovelar et al., 2024). Thus, in older animals, a larger fecal sample volume would allow for a more accurate diagnosis. The Ueno (Ueno & Gonçalves, 1998) and modified Rugai et al. (1954) techniques are sensitive to *D. viviparus* diagnosis; however, animal age information is necessary to determine the most applicable technique.

By evaluating the parameters of storage time, sedimentation time, and water temperature for the recovery of *D. viviparus* L1 larvae, we can observe that shorter storage time combined with water heating and longer sedimentation time optimize diagnosis with a higher LPG count. However, for a faster diagnosis, it would be advisable to perform the techniques with water heated to 46 ± 1°C and an evaluation time of two hours. But in laboratories where it is not possible to certify that the water is heated to the correct temperature, a 12 hour sedimentation time is recommended for a more accurate diagnosis. Thus, the choice of which technique to use, which sedimentation period, whether or not to heat the water, should take into account the reality of the diagnostic laboratory and the origin of the samples, such as the distance from the collection site to the examination site, the housing conditions of the samples until they arrive at the laboratory, the age of the animals, and their clinical condition, since animals with higher parasite loads tend to present more severe clinical symptoms.

In conclusion, both the Ueno (Ueno & Gonçalves, 1998) and the modified Rugai et al. (1954) techniques proved efficient for *D. viviparus* L1 detection, with their sensitivity being influenced by sample sedimentation time and water temperature, while the number of detected larvae was not significantly affected by the sample refrigeration time.

## Data Availability

Data will be made available on request.
